# Matching-adjusted indirect comparison of kidney function in patients with immunoglobulin A nephropathy treated with nefecon or sparsentan

**DOI:** 10.57264/cer-2025-0045

**Published:** 2025-11-20

**Authors:** Christopher Ngai, Mit Patel, Agnieszka Kopiec, Shuai Fu, Noemi Hummel

**Affiliations:** 1 Calliditas Therapeutics, New York, NY 10017, USA; 2 Certara, Kraków, Poland; 3 Huaiyin Institute of Technology, Huai'an, Jiangsu Province, China; 4 Certara GmbH, Lörrach, Germany

**Keywords:** immunoglobulin A nephropathy, kidney failure, matching adjusted indirect comparison, nefecon, sparsentan

## Abstract

**Aim:**

We compared the effects of nefecon, an oral targeted-release budesonide formulation, and sparsentan, an oral, dual endothelin-angiotensin receptor antagonist, on estimated glomerular filtration rate (eGFR) in patients with immunoglobulin A nephropathy, a leading cause of chronic kidney disease.

**Materials & methods:**

We conducted an anchored matching-adjusted indirect comparison (MAIC) using patient-level data from NefIgArd (NCT03643965; n = 364), a randomized (1:1) trial of nefecon plus optimized renin–angiotensin system inhibitor (RASi) therapy versus placebo plus RASi; and aggregate data from PROTECT (NCT03762850; n = 404), a randomized (1:1) trial of sparsentan versus irbesartan, an angiotensin receptor blocker. Mean absolute eGFR change and mean relative urine protein-to-creatinine and urine albumin-to-creatinine ratio changes from baseline at 9, 12 and 24 months (NefIgArd) or 36, 48 and 106 weeks (PROTECT) were analyzed using a mixed-effects model for repeated measures. A composite outcome (time to confirmed 40% eGFR reduction, end-stage kidney disease or all-cause mortality) was also included. An unanchored MAIC and network meta-analysis were used as sensitivity analyses.

**Results:**

The matching process reduced the effective sample for the NefIgArd trial from 364 to 208. Absolute eGFR change significantly favored nefecon over sparsentan at 9 months (mean difference, ml/min/1.73 m^2^ [95% credible interval]: 5.7 [3.1–8.2]), 12 months (3.5 [1.0–6.0]) and 24 months (3.3 [0.0–6.5]). Differences in other outcomes were generally not statistically significant. Sensitivity analysis results were consistent with the main findings.

**Conclusion:**

In patients with immunoglobulin A nephropathy, nefecon plus optimized RASi may preserve kidney function to a greater extent than sparsentan.

## Plain language summary

**What is this article about?:**

Immunoglobulin A nephropathy (IgAN) is a progressive form of kidney disease that can lead to kidney failure in many patients over time. In recent years, different treatments have been approved for use in patients with IgAN, but no head-to-head comparisons of their effectiveness have been conducted. Nefecon is a targeted-release formulation of budesonide that targets the main underlying cause of IgAN in the gut. Sparsentan works downstream by reducing damage to the kidney. We carried out an anchored matching-adjusted indirect comparison of these new treatments using available data from their two pivotal phase III clinical trials (NefIgArd and PROTECT) to compare their effects on kidney function.

**What were the results?:**

Nefecon on top of existing standard of care, was associated with less of a decline in estimated glomerular filtration rate (a measure of kidney function) at 9, 12 and 24 months than sparsentan. We carried out other similar analyses that supported this finding.

**What do the results of the study mean?:**

The results suggest that nefecon is better at preserving kidney function than sparsentan. However, it is important to remember that they work in different ways and so potentially can be used as part of a wider strategy to prevent patients with IgAN from progressing to kidney failure.

## Shareable abstract

This is a matching-adjusted indirect comparison of the efficacy profiles of two approved therapies (nefecon and sparsentan) for immunoglobulin A nephropathy using phase III clinical trial data. Nefecon (plus standard of care) preserved kidney function better than sparsentan.

Immunoglobulin A (IgA) nephropathy (IgAN) is the most common form of glomerular disease globally and a leading cause of chronic kidney disease [1]. It is estimated that 50–75% of patients with IgAN develop kidney failure within 20 years of diagnosis [1]. In recent years, it has been shown that even patients previously regarded as being at ‘low risk’ (i.e., time-averaged proteinuria <0.88 g/gram) have high rates of kidney failure within 10 years [2]. IgAN is thought to be caused by high levels of aberrantly glycosylated IgA in the circulation, which results in the formation of IgA1-IgG/IgA-containing immune complexes that are deposited in the glomeruli and trigger kidney damage [1,3]. Historically, treatment has centered on supportive care, including long-term renin–angiotensin system inhibitor (RASi) therapy [4].

Nefecon is an oral, targeted-release formulation of the corticosteroid budesonide specifically designed to deliver the active drug to the distal ileum [5], a key location for aberrant IgA production [3]. Nefecon was the first agent to receive conditional, accelerated US FDA approval for the treatment of IgAN in December 2021 [6]. In December 2023, the FDA granted full approval of nefecon to reduce the loss of kidney function in adults with primary IgAN who are at risk of disease progression [7,8], based on 2-year data from the phase III NefIgArd trial [9]. Nefecon has also been approved for use in patients with IgAN in the European Union, China and other territories globally [10,11].

Sparsentan is an oral, dual endothelin-angiotensin receptor antagonist intended to reduce damage in the kidney resulting from increased endothelin-1 expression seen in patients with IgAN [12]. It was the second agent to receive conditional, accelerated FDA approval for use in patients with IgAN in February 2023 [13]. In September 2024, sparsentan was granted full approval by the FDA to slow kidney function decline in adults with primary IgAN who are at risk of disease progression, based on the results of the phase III PROTECT study [14,15]. It has also been granted standard marketing authorization in the European Union and temporary approval in Switzerland [16,17].

No head-to-head studies comparing nefecon and sparsentan have been conducted. In addition, enrollment criteria for NefIgArd [9] and PROTECT [15] were sufficiently different to preclude comparison between the two agents using a standard network meta-analysis, which relies on the assumption of homogeneous trial populations [18]. For example, estimated glomerular filtration rate (eGFR), age, sex and racial characteristics (all baseline characteristics that differed between NefIgArd [9] and PROTECT [15]) have been identified as predictors of IgAN progression [19,20] and should be adjusted for. Matching-adjusted indirect comparison (MAIC) is a widely accepted methodology for comparing treatments across trials in the absence of head-to-head comparisons and when adjustment for differences in baseline patient characteristics is required [18].

In this analysis, we used MAIC to compare the effects of nefecon (in combination with optimized RASi treatment) and sparsentan on kidney function in patients with IgAN.

## Materials & methods

### Trials & patients

In the NefIgArd trial (NCT03643965), adult patients with biopsy-confirmed primary IgAN, eGFR 35–90 ml/min/1.73 m^2^ and proteinuria (urine protein-to-creatinine ratio [UPCR] ≥0.8 g/g or ≥1 g/24 h) despite optimized RASi standard of care received nefecon 16 mg/day plus optimized RASi, or placebo plus optimized RASi (i.e., standard of care alone) for 9 months, followed by 15 months of optimized RASi alone [9]. In PROTECT (NCT03762850), adult patients with biopsy-confirmed primary IgAN, eGFR ≥30 ml/min/1.73 m^2^ and proteinuria (≥1 g/24 h) despite optimized RASi standard of care received continuous treatment with sparsentan (target dose 400 mg/day) or irbesartan (target dose 300 mg/day), an angiotensin receptor blocker, for 110 weeks, followed by a 4-week follow-up period on standard of care alone [15,21].

We used individual patient-level data from NefIgArd and aggregate data from the report on PROTECT baseline characteristics [21], PROTECT prespecified interim analysis [22], PROTECT full 2-year analysis [15], and presented data from another MAIC of sparsentan versus nefecon [23,24].

### Efficacy outcomes

In NefIgArd [9], nefecon treatment effects were expressed as changes from baseline at months 9, 12 and 24, which were compared with the effects of sparsentan at the approximately corresponding weeks 36, 48 and 106. Both anchored and unanchored MAICs were performed for the mean absolute change in eGFR from baseline at 9, 12 and 24 months, mean relative changes in UPCR and urine albumin-to-creatinine ratio (UACR) from baseline at 9, 12 and 24 months and a composite outcome consisting of the time to confirmed 40% eGFR reduction, end-stage kidney disease (ESKD), or all-cause mortality (Table 1).

**Table 1.** Matching-adjusted indirect comparisons performed.

End point	Time points from NefIgArd (vs time points from PROTECT)
eGFR LS mean change from baseline	Month 9 vs week 36
UPCR geometric LS mean percent change from baseline	Month 12 vs week 48
UACR geometric LS mean percent change from baseline	Month 24 vs week 106 (closest time point to month 24)
Time to confirmed 40% eGFR reduction, ESKD or all-cause mortality	N/A

eGFR: Estimated glomerular filtration rate; ESKD: End-stage kidney disease; LS: Least-squares; N/A: Not applicable; UACR: Urine albumin-to-creatinine ratio; UPCR: Urine protein-to-creatinine ratio.

### MAIC approach

**Figure 1.**
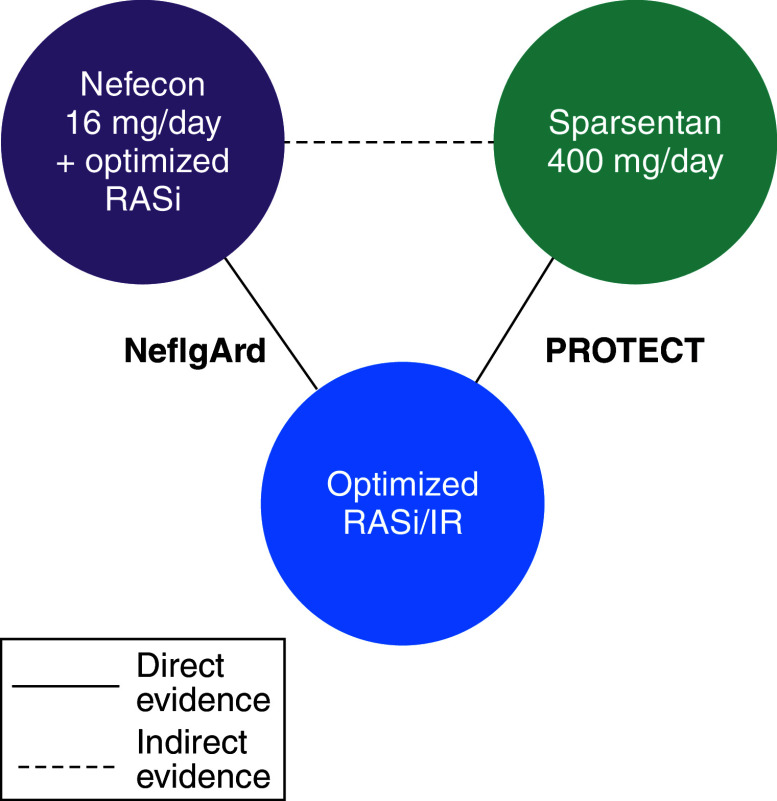
Network for anchored matching-adjusted indirect comparison (all end points). IR: Irbesartan; RASi: Renin–angiotensin system inhibitor.

Our main analytical approach was the MAIC, performed to estimate the effects of nefecon (plus optimized RASi) and sparsentan on different parameters of renal function (Figure 1). The MAIC methodology applies weights to individual patient data from one trial (in this case, NefIgArd) so that baseline characteristics align with published aggregate data from another trial (in this case, PROTECT), thereby reducing bias in the indirect comparison. To differentiate between anchored and unanchored MAIC, in an anchored MAIC the treatments are compared through a common comparator (e.g., placebo or standard of care), whereas in an unanchored MAIC no common comparator exists, and the comparison relies entirely on adjusting for differences in baseline characteristics between the two treatment arms. The anchored MAIC was chosen because the control arms (placebo plus RASi [NefIgArd] or irbesartan [PROTECT]) were considered similar enough due to irbesartan being a RASi and so could be used as common comparators. The use of anchoring via common comparators should make the estimates less biased by the existence of unbalanced prognostic variables [25,26]. An unanchored MAIC was also performed as a sensitivity analysis.

Baseline characteristics used as matching variables included age (years), male sex (%), White race (%), eGFR (ml/min/1.73 m^2^), UPCR (g/g), patients with urinary protein excretion >1.8 g/day (%) and patients with UACR (%) above a prespecified threshold. The threshold was 1.1 g/g when matching overall NefIgArd to the overall PROTECT dataset (anchored MAIC) and when matching the placebo arm from NefIgArd to the irbesartan arm from PROTECT (unanchored MAIC), and was 1.0 g/g when matching the nefecon arm from NefIgArd to the sparsentan arm from PROTECT (unanchored MAIC). All matching covariates were used in the analysis of each end point, unless stated otherwise (Supplementary Table 1). The matching variables were selected based on the knowledge of common risk factors for IgAN progression [19], on clinical experience, and on the availability of the data used.

In the anchored analysis, propensity weights were applied to individual patient data in the active and control arms of the NefIgArd study so that the overall NefIgArd population matched the overall population of the PROTECT study. In the unanchored analysis, propensity weights were applied to individual patient data in the active and control arms of NefIgArd separately, to match baseline characteristics in the corresponding arms of PROTECT.

Absolute changes in eGFR over time and in the log-transformed postbaseline to baseline ratios in UPCR and UACR were estimated using a mixed-effects model for repeated measures, with treatment group, visit, baseline eGFR or log-transformed baseline UPCR (or UACR) values, baseline eGFR or log-transformed baseline UPCR or UACR values with visit interaction, and treatment group with visit interaction as covariates. Time to confirmed 40% eGFR reduction, ESKD, or all-cause mortality was estimated using a Cox proportional hazard model.

In the anchored MAIC, a Bayesian fixed-effects network meta-analysis was performed on the relative effect from PROTECT and the weighted relative effect from NefIgArd, to derive mean differences or geometric mean ratios at individual visits or a hazard ratio (HR), with the associated 95% credible intervals (CrIs), using the R package GeMTC (version 1.0–2) available under a general public license [27–31]. In the unanchored MAIC, arm-level weighted results from NefIgArd were compared with each other to derive mean differences or geometric mean ratios at individual visits or an HR, with the associated 95% confidence intervals.

A standard fixed-effects network meta-analysis model was also applied as a sensitivity analysis. The methods employed for this analysis are described separately in the Supplementary Methods & Supplementary Table 2.

## Results

Data sources for each matching variable in the PROTECT trial are summarized in Supplementary Table 1. In each case, the source providing the best evidence was chosen. For example, means were preferred over medians, and numerals with more digits after the decimal place were preferred over those with fewer digits.

In the matching process, the effective sample size for the NefIgArd trial was reduced from 364 to 208, versus 404 patients in the PROTECT trial (Table 2), which was considered acceptable. The distribution of weights derived for the anchored MAIC is shown in Supplementary Figure 1.

**Table 2.** Anchored matching-adjusted indirect comparison: matching-adjustment of the NefIgArd and PROTECT trial populations.

Parameter	NefIgArd	PROTECT	Weighted NefIgArd
n	364	404	208^†^
Age (years)	42.70	46.00	46.00
Male (%)	65.93	69.80	69.80
White (%)	75.55	67.33	67.33
Mean eGFR (ml/min/1.73 m^2^)	57.87	56.95	56.95
Mean UPCR (g/g)	1.48	1.44	1.44
UACR >1.1 g/g (%)	40.66	50.00	50.00
Urinary protein excretion >1.8 g/day (%)	65.66	50.00	50.00

† Number shown is the effective sample size after weighting.

eGFR: Estimated glomerular filtration rate; UACR: Urine albumin-to-creatinine ratio; UPCR: Urine protein-to-creatinine ratio.

**Figure 2.**
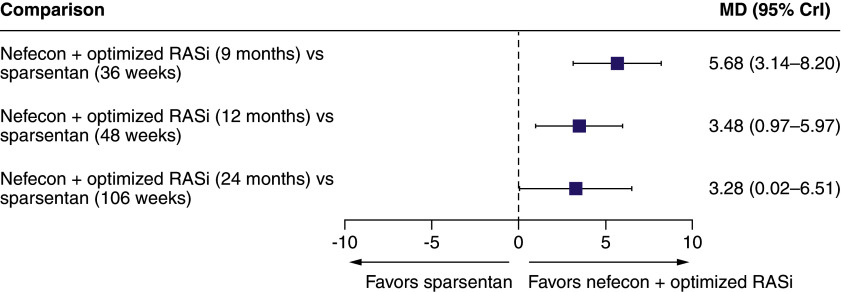
Forest plot for the anchored matching-adjusted indirect comparison for estimated glomerular filtration rate at different time points. CrI: Credible interval; MD: Mean difference; RASi: Renin–angiotensin system inhibitor.

The mean absolute changes in eGFR from baseline for nefecon and placebo in NefIgArd before and after matching to PROTECT were similar (data not shown). There was a statistically significant mean (95% CrI) difference in the absolute change in eGFR favoring nefecon versus sparsentan of 5.68 (3.14–8.20) ml/min/1.73 m^2^ at 9 months (36 weeks), 3.48 (0.97–5.97) ml/min/1.73 m^2^ at 12 months (48 weeks) and 3.28 (0.02–6.51) ml/min/1.73 m^2^ at 24 months (106 weeks) from the anchored MAIC (Figure 2).

**Figure 3.**
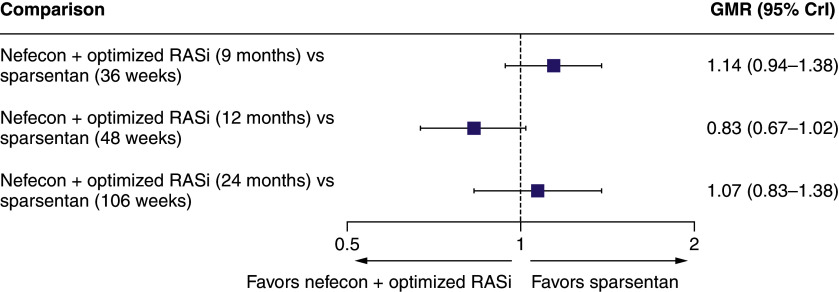
Forest plot for the anchored matching-adjusted indirect comparison for urine protein-to-creatinine ratio at different time points. CrI: Credible interval; GMR: Geometric mean ratio; RASi: Renin–angiotensin system inhibitor.

**Figure 4.**
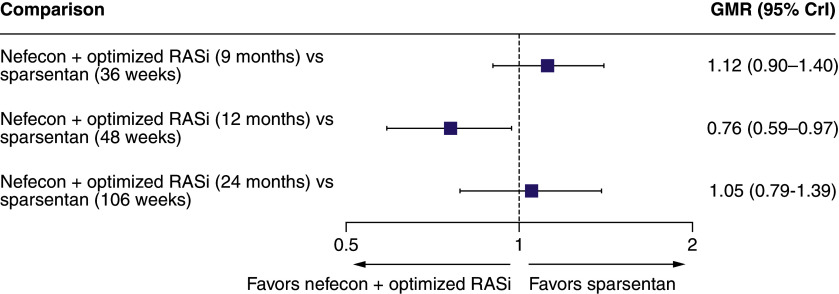
Forest plot for the anchored matching-adjusted indirect comparison for urine albumin-to-creatinine ratio at different time points. CrI: Credible interval; GMR: Geometric mean ratio; RASi: Renin–angiotensin system inhibitor.

There were no significant differences in the UPCR geometric mean ratios between nefecon and sparsentan at any time point included in the anchored MAIC analysis (Figure 3). For UACR, there was a statistically significant difference in favor of nefecon at 12 months (48 weeks) but not at the other time points from the anchored MAIC (Figure 4). There was no significant difference between nefecon and sparsentan in time to confirmed 40% eGFR reduction, ESKD, or all-cause mortality from the anchored MAIC (HR [95% CrI]: 0.85 [0.31–2.28]).

In the sensitivity analysis using unanchored MAIC, the effective sample size was 132 in the nefecon arm of NefIgArd versus 202 patients in the PROTECT sparsentan arm, and 94 in the NefIgArd placebo arm versus 202 patients in the PROTECT irbesartan arm (Supplementary Table 3). The distributions of weights derived for the unanchored MAIC are shown in Supplementary Figure 2. Efficacy assessments were directionally consistent with those obtained using anchored MAIC (Supplementary Figure 3).

The results of the standard fixed-effects network meta-analysis were also directionally consistent with those of the MAIC analyses, with mean differences in the change in eGFR from baseline achieving statistical significance in favor of nefecon versus sparsentan at months 9, 12 and 24 (Supplementary Table 4).

## Discussion

In many countries, both nefecon and sparsentan have now received full approval for use in patients with IgAN. Therefore, a meaningful comparison of their treatment effects is of interest to patients, caregivers, clinicians and payers. After accounting for differences in the patient populations from the NefIgArd and PROTECT trials, thus ensuring transitivity (an important assumption of all network meta-analyses), our analysis showed that, at 2 years, treatment with nefecon 16 mg/day plus optimized RASi for 9 months (followed by 15 months off treatment) was associated with a greater eGFR benefit compared with continuous treatment with sparsentan 400 mg/day over 2 years. Significant differences were observable at 9 months when treatment with nefecon was stopped, and these significant differences persisted at 12 and 24 months, when patients in the NefIgArd trial had completed a further 15 months on optimized RASi only. Since eGFR is a direct marker of kidney function, this suggests that nefecon has a greater impact than sparsentan on the pathophysiologic origin of IgAN. *Post hoc* power estimates for the Bayesian network meta-analysis [32] of the weighted NefIgArd and PROTECT trials for eGFR at month 9/week 36, month 12/week 48 and month 24/week 106 were 88%, 58% and 45%, respectively, supporting the generalizability of these results.

The other efficacy parameters were not statistically different between the two treatments, except for a UACR advantage of nefecon at 12 months (48 weeks), which may reflect the fact that, in the NefIgArd trial, the greatest reductions in both UPCR and UACR with nefecon were measured 3 months after the 9-month treatment period had been completed. Similarity between the findings obtained using anchored and unanchored MAIC and standard network meta-analysis supports the robustness of our findings.

The findings of other (unanchored) MAIC analyses comparing nefecon with sparsentan have been presented elsewhere [23,24,33]. These analyses have reported significantly greater mean percentage UPCR reductions from baseline at both 9 months and 2 years with sparsentan versus nefecon. The 2-year analysis of difference in eGFR total slope also yielded a different result from the current analysis, claiming a ‘slower decline in kidney function’ with sparsentan versus nefecon, although this was not statistically significant [33]. In the current analysis, the UPCR geometric mean ratio at 24 months (106 weeks) also numerically favored sparsentan but did not achieve statistical significance. It is important to note that assessing differences between treatments across different time points versus one single time point provides more informative results, and the current analysis showed statistically significant differences in absolute eGFR change from baseline that favored nefecon over sparsentan. There are also differences in the variables chosen for matching in these unanchored analyses compared with the current anchored analysis, which could explain the differences in their findings. The limitations of using unanchored versus anchored approaches, and the choice of matched variables having a considerable impact on the outcomes of an unanchored MAIC [26], should be taken into consideration when attempting to compare and contrast these different findings.

### Limitations

It should be noted that the treatment optimization approaches differed between the control arms of NefIgArd (RASi) and PROTECT (irbesartan), which may have biased the anchored analysis. However, directionally consistent findings between the anchored and unanchored MAICs suggest that such a bias, if present, had only a limited impact on our analysis. In addition, the MAIC method cannot adjust for unobserved or unobservable effect modifiers, but the baseline characteristics that we used as matching variables (age, sex, race, baseline eGFR, UPCR, urinary protein excretion and UACR) are prognostic factors of disease progression and likely modifiers [19] of the treatment effect. Furthermore, this analysis, like other indirect comparisons, assumes an exchangeability of patients between studies, which cannot be assessed directly. Finally, there are disadvantages associated with the use of a composite end point: for example, all component end points we selected are typically late occurring and therefore less likely to be captured during the short trial follow-up. Additionally, each component (time to confirmed 40% eGFR reduction, ESKD or all-cause mortality) was assigned equal importance, which may not reflect the typical disease progression. As a result, the composite end point was likely not a sensitive measure of efficacy [34].

This analysis also does not take into account the safety profiles of the two agents. In the NefIgArd trial, peripheral edema, hypertension, muscle spasms, acne and headache were the most commonly reported treatment-emergent adverse events with nefecon and standard of care versus standard of care alone; these were generally nonserious and mild in nature, and were reversible during or after treatment [9]. In PROTECT, dizziness and hypotension were among treatment-emergent adverse events that were reported to occur more frequently with sparsentan than with active control [15]. These may be factors that clinicians take into account when treating patients; however, the purpose of the current analysis was to provide an objective comparison of kidney function as opposed to safety measures. Furthermore, nefecon was given alongside optimized RASi in NefIgArd [9], whereas irbesartan (also a RASi) was the comparator to sparsentan in PROTECT [15]. As a result, a formal indirect treatment comparison of the safety profiles of nefecon and sparsentan in these trials would be confounded by this.

## Conclusions

The eGFR results from our MAIC analyses suggest that nefecon plus optimized RASi may preserve kidney function to a greater extent than sparsentan for the 9 months when patients were on nefecon treatment. However, the gap in eGFR preservation appears to narrow during the off-treatment period with nefecon, suggesting that there is a benefit with continuous sparsentan treatment. It is important to note that the two agents should not be considered as mutually exclusive treatment options. The updated Kidney Disease: Improving Global Outcomes 2025 guidelines [35] advocate for a ‘two-pronged’ approach to the management of IgAN wherein the focus is to “*simultaneously prevent or reduce IgA-containing immune complex formation and IgA-containing immune complex-mediated glomerular injury” and “manage the consequences of existing IgAN-induced nephron loss.*” In this context, nefecon and sparsentan belong to the first and second ‘prongs’, respectively. In these updated guidelines, nefecon is described as “the only treatment to date proven to reduce the levels of pathogenic forms of IgA and IgA-containing immune complexes,” and a 9-month course is suggested for patients who are at risk of progressive loss of kidney function with IgAN [35]. Our findings provide additional support for nefecon as a disease-modifying therapy and a cornerstone component of any IgAN management strategy. More recently, iptacopan, a complement factor B inhibitor and atrasentan, a selective endothelin A receptor antagonist, have received conditional, accelerated FDA approval in patients with IgAN [36–39]; further comparative analyses are planned when more data for these agents become available.

## Summary points

- Immunoglobulin A nephropathy is a chronic progressive glomerular disease that can lead to kidney failure in many patients.
- Two approved therapies for immunoglobulin A nephropathy, nefecon (a targeted-release formulation of budesonide) and sparsentan (a dual endothelin-angiotensin receptor antagonist), have been shown to preserve kidney function, as measured by a reduced decline in estimated glomerular filtration rate (eGFR).
- Matching-adjusted indirect comparison is an increasingly popular method for efficacy assessments when head-to-head trials are not available and when network meta-analyses are not feasible due to heterogeneous patient populations.
- Our anchored matching-adjusted indirect comparison analysis was based on data from randomized phase III trials of nefecon (NefIgArd; NCT03643965) and sparsentan (PROTECT; NCT03762850).
- Mean absolute change in eGFR and mean relative changes in urine protein- and urine albumin-to-creatinine ratios from baseline at 9, 12 and 24 months following treatment initiation were assessed.
- The analysis reported an absolute change in eGFR that significantly favored nefecon plus standard of care over sparsentan at all three time points. Sensitivity analyses supported these findings.
- Differences in other outcomes were generally not statistically significant.
- This finding suggests that nefecon works differently from sparsentan in reducing eGFR decline.

## Supplementary Material



## References

[1] Cheung CK, Alexander S, Reich HN The pathogenesis of IgA nephropathy and implications for treatment. Nat. Rev. Nephrol. 21(1), 9–23 (2025). 39232245 10.1038/s41581-024-00885-3 PMC7616674

[2] Pitcher D, Braddon F, Hendry B Long-term outcomes in IgA nephropathy. Clin. J. Am. Soc. Nephrol. 18(6), 727–738 (2023). 37055195 10.2215/CJN.0000000000000135 PMC10278810

[3] Barratt J, Rovin BH, Cattran D Why target the gut to treat IgA nephropathy? Kidney Int. Rep. 5(10), 1620–1624 (2020). 33102954 10.1016/j.ekir.2020.08.009 PMC7569689

[4] Kidney Disease: Improving Global Outcomes Glomerular Diseases Work Group. KDIGO 2021 clinical practice guideline for the management of glomerular diseases. Kidney Int. 100(Suppl. 4), S1–S276 (2021). 34556256 10.1016/j.kint.2021.05.021

[5] Barratt J, Kristensen J, Pedersen C, Jerling M. Insights on Nefecon^®^, a targeted-release formulation of budesonide and its selective immunomodulatory effects in patients with IgA nephropathy. Drug Des. Devel. Ther. 18, 3415–3428 (2024). 10.2147/DDDT.S383138 PMC11298173 39100224

[6] PR Newswire. FDA grants Calliditas Therapeutics accelerated approval of TARPEYO™ (budesonide) to reduce proteinuria in IgA nephropathy. (Accessed: 19 September 2025). https://www.prnewswire.com/news-releases/fda-grants-calliditas-therapeutics-accelerated-approval-of-tarpeyo-budesonide-to-reduce-proteinuria-in-iga-nephropathy-301445918.html

[7] PR Newswire. Everest Medicines' partner Calliditas Therapeutics announces Nefecon^®^ the only FDA-approved treatment for IgA nephropathy to significantly slow kidney function decline. (Accessed: 19 September 2025). https://www.prnewswire.com/news-releases/everest-medicines-partner-calliditas-therapeutics-announces-nefecon-the-only-fda-approved-treatment-for-iga-nephropathy-to-significantly-slow-kidney-function-decline-302020586.html

[8] Calliditas Therapeutics AB. Tarpeyo^®^ (targeted release formulation budesonide) US Food and Drug Administration. US Prescribing Information. (2024). https://www.tarpeyo.com/prescribinginformation.pdf

[9] Lafayette R, Kristensen J, Stone A Efficacy and safety of a targeted-release formulation of budesonide in patients with primary IgA nephropathy (NefIgArd): 2-year results from a randomised phase III trial. Lancet 402(10405), 859–870 (2023). 37591292 10.1016/S0140-6736(23)01554-4

[10] STADA Arzneimittel AG. Kinpeygo (budesonide) European Summary of Product Characteristics. (2023). https://www.ema.europa.eu/documents/product-information/kinpeygo-epar-product-information_en.pdf

[11] Zhou W. Nefecon approved for use in China. (Accessed: 19 September 2025). https://global.chinadaily.com.cn/a/202311/28/WS65655deda31090682a5f0630.html#

[12] Kohan DE, Bedard PW, Jenkinson C, Hendry B, Komers R. Mechanism of protective actions of sparsentan in the kidney: lessons from studies in models of chronic kidney disease. Clin. Sci. (Lond.) 138(11), 645–662 (2024). 38808486 10.1042/CS20240249 PMC11139641

[13] Travere Therapeutics Inc. Travere therapeutics announces FDA accelerated approval of FILSPARI™ (sparsentan), the first and only non-immunosuppressive therapy for the reduction of proteinuria in IgAN nephropathy. (Accessed: 19 September 2025). https://ir.travere.com/news-releases/news-release-details/travere-therapeutics-announces-fda-accelerated-approval

[14] Travere Therapeutics Inc. Travere Therapeutics announces full FDA approval of FILSPARI^®^ (sparsentan), the only non-immunosuppressive treatment that significantly slows kidney function decline in IgA nephropathy. (Accessed: 19 September 2025). https://ir.travere.com/news-releases/news-release-details/travere-therapeutics-announces-full-fda-approval-filsparir

[15] Rovin BH, Barratt J, Heerspink HJL Efficacy and safety of sparsentan versus irbesartan in patients with IgA nephropathy (PROTECT): 2-year results from a randomised, active-controlled, phase III trial. Lancet 402(10417), 2077–2090 (2023). 37931634 10.1016/S0140-6736(23)02302-4

[16] Travere Therapeutics Inc. Travere Therapeutics and CSL Vifor Announce Standard EU Approval of FILSPARI^®^ (sparsentan) for IgA Nephropathy. (Accessed: 19 September 2025). https://ir.travere.com/press-releases/news-details/2025/Travere-Therapeutics-and-CSL-Vifor-Announce-Standard-EU-Approval-of-FILSPARI-sparsentan-for-IgA-Nephropathy/default.aspx

[17] Travere Therapeutics Inc. Travere Therapeutics and CSL Vifor announce Swissmedic approval of FILSPARI^®^ (sparsentan) for the treatment of IgA nephropathy. (Accessed: 19 September 2025). https://ir.travere.com/news-releases/news-release-details/travere-therapeutics-and-csl-vifor-announce-swissmedic-approval

[18] Macabeo B, Quenéchdu A, Aballéa S Methods for indirect treatment comparison: results from a systematic literature review. J. Mark. Access Health Policy 12(2), 58–80 (2024). 38660413 10.3390/jmahp12020006 PMC11036291

[19] Barbour SJ, Coppo R, Zhang H Evaluating a new international risk-prediction tool in IgA nephropathy. JAMA Intern. Med. 179(7), 942–952 (2019). 30980653 10.1001/jamainternmed.2019.0600 PMC6583088

[20] O'Shaughnessy MM, Hogan SL, Thompson BD Glomerular disease frequencies by race, sex and region: results from the International Kidney Biopsy Survey. Nephrol. Dial. Transplant. 33(4), 661–669 (2018). 29106637 10.1093/ndt/gfx189 PMC6659026

[21] Barratt J, Rovin B, Wong MG IgA nephropathy patient baseline characteristics in the sparsentan PROTECT study. Kidney Int. Rep. 8(5), 1043–1056 (2023). 37180506 10.1016/j.ekir.2023.02.1086 PMC10166729

[22] Heerspink HJL, Radhakrishnan J, Alpers CE Sparsentan in patients with IgA nephropathy: a prespecified interim analysis from a randomised, double-blind, active-controlled clinical trial. Lancet 401(10388), 1584–1594 (2023). 37015244 10.1016/S0140-6736(23)00569-X

[23] Chai X, Bensink M, Gao S #4499 Matching-adjusted indirect comparison of sparsentan vs delayed-release formulation budesonide for proteinuria reduction in adults with IgA nephropathy. Nephrol. Dial. Transplant. 38, i395 (2023).

[24] Bensink M, Gong W, Chai X Matching-adjusted indirect comparison of sparsentan vs. delayed-release formulation budesonide for proteinuria reduction in adults with IgA nephropathy. Presented at: 60th European Renal Association Congress. Milan, Italy (15–18 June 2023). (Accessed: 19 September 2025). https://medicalaffairs.travere.com/wp-content/uploads/2023/10/2023_ERA_Bensink_MAIC-Spar-v-Budesodine_Focused-Oral-pdf.pdf

[25] Jiang Z, Cappelleri JC, Gamalo M A comprehensive review and shiny application on the matching-adjusted indirect comparison. Res. Synth. Methods 15(4), 671–686 (2024). 38380799 10.1002/jrsm.1709

[26] Phillippo DM, Ades AE, Dias S Methods for population-adjusted indirect comparisons in health technology appraisal. Med. Decis. Making 38(2), 200–211 (2018). 28823204 10.1177/0272989X17725740 PMC5774635

[27] van Valkenhoef G, Dias S, Ades AE, Welton NJ. Automated generation of node-splitting models for assessment of inconsistency in network meta-analysis. Res. Synth. Methods 7(1), 80–93 (2016). 26461181 10.1002/jrsm.1167 PMC5057346

[28] van Valkenhoef G, Luzqz G, de Brock B Automating network meta-analysis. Res. Synth. Methods 3(4), 285–299 (2012). 26053422 10.1002/jrsm.1054

[29] van Valkenhoef G, Kuiper J. GeMTC: network meta-analysis using bayesian methods, version 1.0–2. (Accessed: 19 September 2025). https://cran.r-project.org/web/packages/gemtc/index.html

[30] van Valkenhoef G, Bujkiewicz S, Efthimiou O GeMTC Manual. (Accessed: 19 September 2025). https://gemtc.drugis.org/manual.html

[31] The R Project. The R project for statistical computing. (Accessed: 19 September 2025). https://www.r-project.org/

[32] Thorlund K, Mills EJ. Sample size and power considerations in network meta-analysis. Syst. Rev. 1, 41 (2012). 22992327 10.1186/2046-4053-1-41 PMC3514119

[33] Gong W, Diva U, Bensink M PROTECT and NefIgArd two-year proteinuria and eGFR outcomes in adults with IgA nephropathy: matching-adjusted indirect comparison. Nephrol. Dial. Transplant. 39, i744–i1745 (2024).

[34] Little DJ, Gasparyan SB, Schloemer P Validity and utility of a hierarchical composite end point for clinical trials of kidney disease progression: a review. J. Am. Soc. Nephrol. 34(12), 1928–1935 (2023). 37807165 10.1681/ASN.0000000000000244 PMC10703071

[35] Kidney Disease: Improving Global Outcomes (KDIGO) IgAN and IgAV Work Group. KDIGO 2025 clinical practice guideline for the management of immunoglobulin A nephropathy (IgAN) and immunoglobulin A vasculitis (IgAV). Kidney Int. 108(Suppl. 4), S1–S71 (2025). 40975564 10.1016/j.kint.2025.04.004

[36] Novartis Pharmaceuticals. Novartis receives FDA accelerated approval for Fabhalta^®^ (iptacopan), the first and only complement inhibitor for the reduction of proteinuria in primary IgA nephropathy (IgAN). (Accessed: 19 September 2025). https://www.novartis.com/news/media-releases/novartis-receives-fda-accelerated-approval-fabhalta-iptacopan-first-and-only-complement-inhibitor-reduction-proteinuria-primary-iga-nephropathy-igan

[37] Novartis Pharmaceuticals. Fabhalta^®^ (iptacopan) US Food and Drug Administration. US Prescribing Information. (2025). https://www.novartis.com/us-en/sites/novartis_us/files/fabhalta.pdf

[38] Novartis Pharmaceuticals. Novartis receives FDA accelerated approval for Vanrafia^®^ (atrasentan), the first and only selective endothelin A receptor antagonist for proteinuria reduction in primary IgA nephropathy (IgAN). (Accessed: 19 September 2025). https://www.novartis.com/news/media-releases/novartis-receives-fda-accelerated-approval-vanrafia-atrasentan-first-and-only-selective-endothelin-receptor-antagonist-proteinuria-reduction-primary-iga-nephropathy-igan

[39] Novartis Pharmaceuticals. Vanrafia^®^ (atrasentan) US Food and Drug Administration. US Prescribing Information. (2025). https://www.novartis.com/us-en/sites/novartis_us/files/vanrafia.pdf

