# Supplementary material for: Matching-adjusted indirect comparison of kidney function in patients with immunoglobulin A nephropathy treated with nefecon or sparsentan
Source: J Comp Eff Res. 2025 Nov 20;14(12):e250045. doi: 10.57264/cer-2025-0045 (PMC12679650; doi:10.57264/cer-2025-0045)
Supplement: Supplementary file 1 [file cer-14-250045-s1.docx]

**Supplementary Material**

**Supplementary methods**

The standard network meta-analysis for nefecon and sparsentan was developed using a fixed-effect model on relative effect data for mean change from baseline [1,2]. Analysis was done in R 4.1.3 using the package *gemtc* and *rjags* to perform the Gibbs sampling procedure for the Bayesian network meta-analysis [3,4].

The model outputs are point estimates and 95% credible intervals between pairs of treatments.

Similar analysis results from the NefIgArd trial on estimated glomerular filtration rate, urine protein-to-creatinine ratio, and urine albumin-to-creatinine ratio as those reported in the PROTECT trial were used as efficacy inputs to the network meta-analysis [5,6]. If similar analysis results were not reported in the NefIgArd publication [5], analysis results were generated using individual patient-level data (**Supplementary Table S2**).

Supplementary Tables and Figures

**Supplementary Table S1.** **Patient baseline characteristics used for the MAICs.**

| **Parameter** | **NefIgArd** | | **PROTECT** | |
| --- | --- | --- | --- | --- |
|  | **Nefecon + optimized RASi** | **Optimized RASi** | **Sparsentan** | **Irbesartan** |
| Patient number | 182 | 182 | 202^†^ | 202^†^ |
| Age (years), mean (SD) | 43.80 (10.78)^‡^ | 41.60 (10.65)^‡^ | 46.56 (12.76)^§^ | 45.43 (12.12)^§^ |
| Male, n (%) | 117 (64.3) | 123 (67.6) | 139^†^ (68.81) | 143^†^ (70.79) |
| White, n (%) | 138 (75.8) | 137 (75.3) | 130^†^ (64.36) | 142^†^ (70.30) |
| eGFR (mL/min/1.73 m^2^), mean (SD) | 58.13 (15.88)^‡^ | 57.61 (15.50)^‡^ | 56.8 (24.3)^†^ | 57.1 (23.6)^†^ |
| UPCR (g/g), median (IQR) | 1.28 (0.90–1.76) | 1.25 (0.88–1.74) | 1.43 (0.9)^§^ | 1.44 (0.89)^§^ |
| UACR (g/g), median (IQR) | 0.99 (0.68–1.40) | 0.98 (0.66–1.42) | 1.0 (0.7–1.5)^¶^ | 1.1 (0.7–1.5)^¶^ |
|  |  |  | Overall: 1.1 (0.7–1.5)^#^ | |
| Urinary protein excretion (g/day), median (IQR) | 2.29 (1.61–3.14) | 2.17 (1.53–3.39) | 1.8 (1.2–2.9)^†^ | 1.8 (1.3–2.6)^†^ |
|  |  |  | Overall: 1.8 (1.3–2.8)^#^ | |

^†^ From Rovin et al. (2023) [6].

^‡^ From individual patient-level data.

^§^ From Bensink et al. (2023) [7], available as mean (SD) instead of median (IQR), or with more digits.

^¶^ From Heerspink et al. (2023) [8]; not available in Rovin et al. (2023) [6].

^#^ From Barratt *et al*. (2023) [9] and used for anchored MAIC only.

eGFR: estimated glomerular filtration rate; IQR: interquartile range; MAIC: matching-adjusted indirect comparison; RASi: renin–angiotensin system inhibitor; SD: standard deviation; UACR: urine albumin-to-creatinine ratio; UPCR: urine protein-to-creatinine ratio.

**Supplementary Table S2.** **Input data for the network meta-analysis.**

| **Endpoint** | **Study** | **Comparison** | **Mean difference in change from baseline** | **95% CI** | **Source** |
| --- | --- | --- | --- | --- | --- |
| **eGFR** | **Month 9/week 36** | | | | |
|  | NefIgArd | Nefecon + optimized RASi vs optimized RASi | 5.53 | 3.49–7.57 | Individual patient-level data^†^ |
|  | PROTECT | Sparsentan vs irbesartan | 0.8 | –0.83 to 2.42 | Rovin *et al*. (2023), Figure 2^‡,§^ |
|  | **Month 12/week 48** | | | | |
|  | NefIgArd | Nefecon + optimized RASi vs optimized RASi | 4.67 | 2.6–6.74 | Individual patient-level data^†^ |
|  | PROTECT | Sparsentan vs irbesartan | 1.52 | –0.08 to 3.13 | Rovin *et al*. (2023), Figure 2^‡,§^ |
|  | **Month 24/week 106** | | | | |
|  | NefIgArd | Nefecon + optimized RASi vs optimized RASi | 5.68 | 3.02–8.34 | Individual patient-level data^†^ |
|  | PROTECT | Sparsentan vs irbesartan | 2.25 | 0.11–4.38 | Rovin *et al*. (2023), Figure 2^‡,§^ |
| **Endpoint** | **Study** | **Comparison** | **Geometric least-squares mean ratio** | **95% CI** | **Source** |
| **UPCR** | **Month 9/week 36** | | | | |
|  | NefIgArd | Nefecon + optimized RASi vs optimized RASi | 0.7 | 0.61–0.8 | Clinical Study Report  (31-May-23), Table 23^†^ |
|  | PROTECT | Sparsentan vs irbesartan | 0.59 | 0.51–0.68 | Rovin *et al*. (2023), Figure 5^‡^ |
|  | **Month 12/week 48** | | | | |
|  | NefIgArd | Nefecon + optimized RASi vs optimized RASi | 0.5 | 0.43–0.58 | Clinical Study Report  (31-May-23), Table 23^†^ |
|  | PROTECT | Sparsentan vs irbesartan | 0.6 | 0.52–0.7 | Rovin *et al*. (2023), Figure 5^‡^ |
|  | **Month 24/week 106** | | | | |
|  | NefIgArd | Nefecon + optimized RASi vs optimized RASi | 0.7 | 0.59–0.83 | Clinical Study Report  (31-May-23), Table 23^†^ |
|  | PROTECT | Sparsentan vs irbesartan | 0.62 | 0.52–0.75 | Rovin *et al*. (2023), Figure 5^‡^ |
| **Endpoint** | **Study** | **Comparison** | **Geometric least-squares mean ratio** | **95% CI** | **Source** |
| **UACR** | **Month 9/week 36** | | | | |
|  | NefIgArd | Nefecon + optimized RASi vs optimized RASi | 0.66 | 0.56–0.77 | Clinical Study Report  (31-May-23), Table 24^†^ |
|  | PROTECT | Sparsentan vs irbesartan | 0.55 | 0.47–0.65 | Rovin *et al*. (2023),  Suppl. Figure 3^‡^ |
|  | **Month 12/week 48** | | | | |
|  | NefIgArd | Nefecon + optimized RASi vs optimized RASi | 0.44 | 0.37–0.53 | Clinical Study Report  (31-May-23), Table 24^†^ |
|  | PROTECT | Sparsentan vs irbesartan | 0.58 | 0.49–0.69 | Rovin *et al*. (2023),  Suppl. Figure 3^‡^ |
|  | **Month 24/week 106** | | | | |
|  | NefIgArd | Nefecon + optimized RASi vs optimized RASi | 0.65 | 0.53–0.79 | Clinical Study Report  (31-May-23), Table 24^†^ |
|  | PROTECT | Sparsentan vs irbesartan | 0.6 | 0.49–0.73 | Rovin *et al*. (2023),  Suppl. Figure 3^‡^ |
| **Endpoint** | **Study** | **Comparison** | **Hazard ratio** | **95% CI** | **Source** |
| **Time to confirmed 40% eGFR reduction, ESKD, or all-cause mortality** | NefIgArd | Nefecon + optimized RASi vs optimized RASi | 0.62 | 0.32–1.21 | Individual patient-level data^¶^ |
|  | PROTECT | Sparsentan vs irbesartan | 0.64 | 0.34–1.18 | Rovin *et al*. (2023), Figure 4^#^ |

^†^ Analyzed using an MMRM including covariates for baseline eGFR, treatment group and visit, and interactions of baseline eGFR with visit, and visit with treatment group. An unstructured covariance matrix was used to model the within-subject correlation of data.

^‡^ Analyzed using an MMRM with fixed effects of treatment group, baseline value in log scale, time (i.e., analysis visit in weeks), treatment group by time interaction, and randomization stratification variable (4 levels based on screening eGFR and urine protein excretion); patient was included as a random effect. An unstructured covariance matrix was used.

^§^ Least-squares mean change from baseline.

^¶^ Analyzed using a Cox proportional hazard model with a fixed-effect term for randomized treatment.

^#^ Kaplan-Meier curves were extracted from the graph; survival data were reconstructed using the method by Guyot *et al*. (2012) [10] and analyzed using a Cox proportional hazard model with a fixed-effect term for randomized treatment.

CI: confidence interval; eGFR: estimated glomerular filtration rate; ESKD: end-stage kidney disease; MMRM: mixed model with repeated measures; RASi: renin–angiotensin system inhibitor; UACR: urine albumin-to-creatinine ratio; UPCR: urine protein-to-creatinine ratio.

**Supplementary Table S3.** **Results from matching-adjustment for the unanchored MAIC.**

| **Parameter** | **Nefecon from NefIgArd** | **Sparsentan from PROTECT** | **Weighted nefecon from NefIgArd** | **Placebo from NefIgArd** | **Irbesartan from PROTECT** | **Weighted placebo from NefIgArd** |
| --- | --- | --- | --- | --- | --- | --- |
| N | 182 | 202 | 132^†^ | 182 | 202 | 94^†^ |
| Age (years) | 43.802 | 46.560 | 46.560 | 41.604 | 45.430 | 45.430 |
| Male (%) | 64.286 | 68.810 | 68.811 | 67.582 | 70.790 | 70.787 |
| White (%) | 75.824 | 64.360 | 64.359 | 75.275 | 70.300 | 70.297 |
| eGFR (mL/min/1.73 m^2^) | 58.134 | 56.800 | 56.800 | 57.607 | 57.100 | 57.100 |
| UPCR (g/g) | 1.484 | 1.430 | 1.430 | 1.484 | 1.440 | 1.440 |
| % with UACR  >1.1 g/g | N/A | N/A | N/A | 39.011 | 50.000 | 50.004 |
| % with UACR  >1.0 g/g | 49.451 | 50.000 | 50.002 | N/A | N/A | N/A |
| % with urinary protein excretion  >1.8 g/day | 65.934 | 50.000 | 50.002 | 65.385 | 50.000 | 50.004 |

^†^ Numbers shown are the ESS after weighting.

eGFR: estimated glomerular filtration rate; ESS: effective sample size; MAIC: matching-adjusted indirect comparison; N/A: not applicable; UACR: urine albumin-to-creatinine ratio; UPCR: urine protein-to-creatinine ratio.

**Supplementary Table S4. Results of the fixed-effects network meta-analysis.**

| **Endpoint** | **Time point** | **Comparison of nefecon + optimized RASi vs sparsentan** |
| --- | --- | --- |
| eGFR, mean difference in change from baseline (95% CrI) | Month 9/week 36 | 4.73 (2.11–7.34)^†^ |
|  | Month 12/week 48 | 3.15 (0.52–5.76)^†^ |
|  | Month 24/week 106 | 3.43 (0.01–6.83)^†^ |
| UPCR, geometric least-squares mean ratio  (95% CrI) | Month 9/week 36 | 1.20 (0.98–1.45) |
|  | Month 12/week 48 | 0.83 (0.68–1.03) |
|  | Month 24/week 106 | 1.12 (0.87–1.45) |
| UACR, geometric least-squares mean ratio  (95% CrI) | Month 9/week 36 | 1.18 (0.94–1.49) |
|  | Month 12/week 48 | 0.77 (0.60–0.98)^†^ |
|  | Month 24/week 106 | 1.09 (0.81–1.45) |
| Time to confirmed 40% eGFR reduction, ESKD, or all-cause mortality, hazard ratio (95% CrI) | N/A | 0.98 (0.39–2.43) |

^†^ Statistically significant.

eGFR mean differences >0 favor nefecon + optimized RASi over sparsentan. UPCR/UACR geometric least-squares mean ratios <1 favor nefecon + optimized RASi over sparsentan. Hazard ratios <1 favor nefecon + optimized RASi over sparsentan.

CrI: credible interval; eGFR: estimated glomerular filtration rate; ESKD: end-stage kidney disease; N/A: not applicable; RASi: renin–angiotensin system inhibitor; UACR: urine albumin-to-creatinine ratio; UPCR: urine protein-to-creatinine ratio.


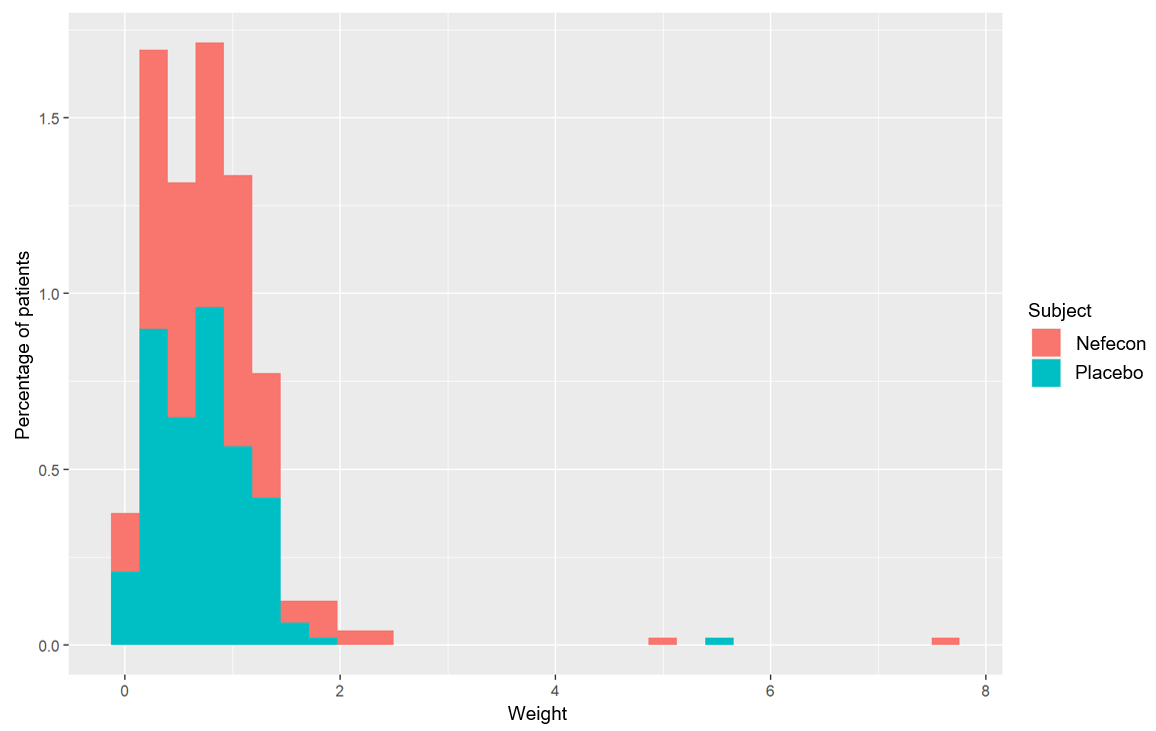


**Supplementary Figure S1.** **Distribution of weights derived for the anchored MAIC.**

MAIC: matching-adjusted indirect comparison.

**A**


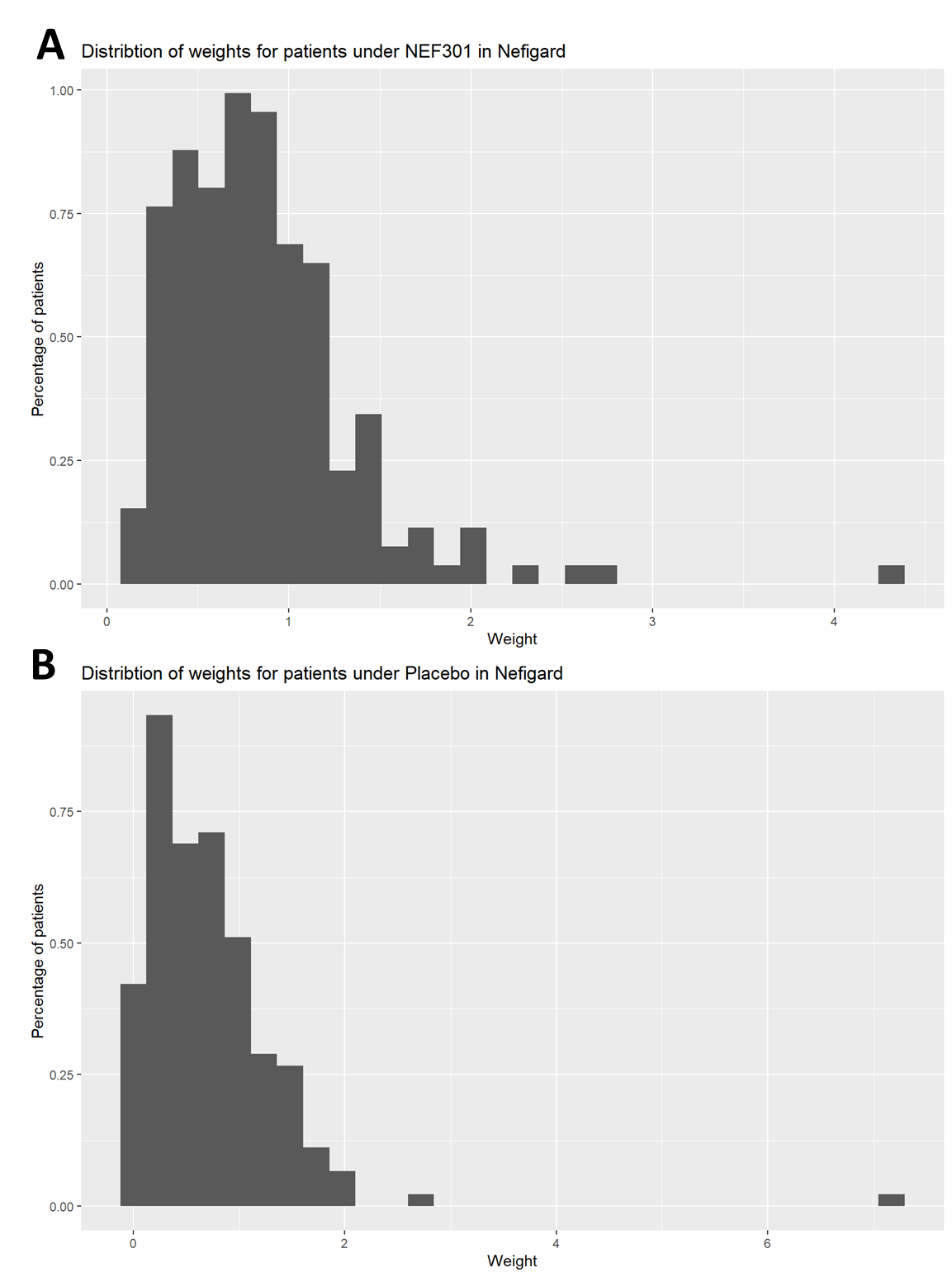


**B**


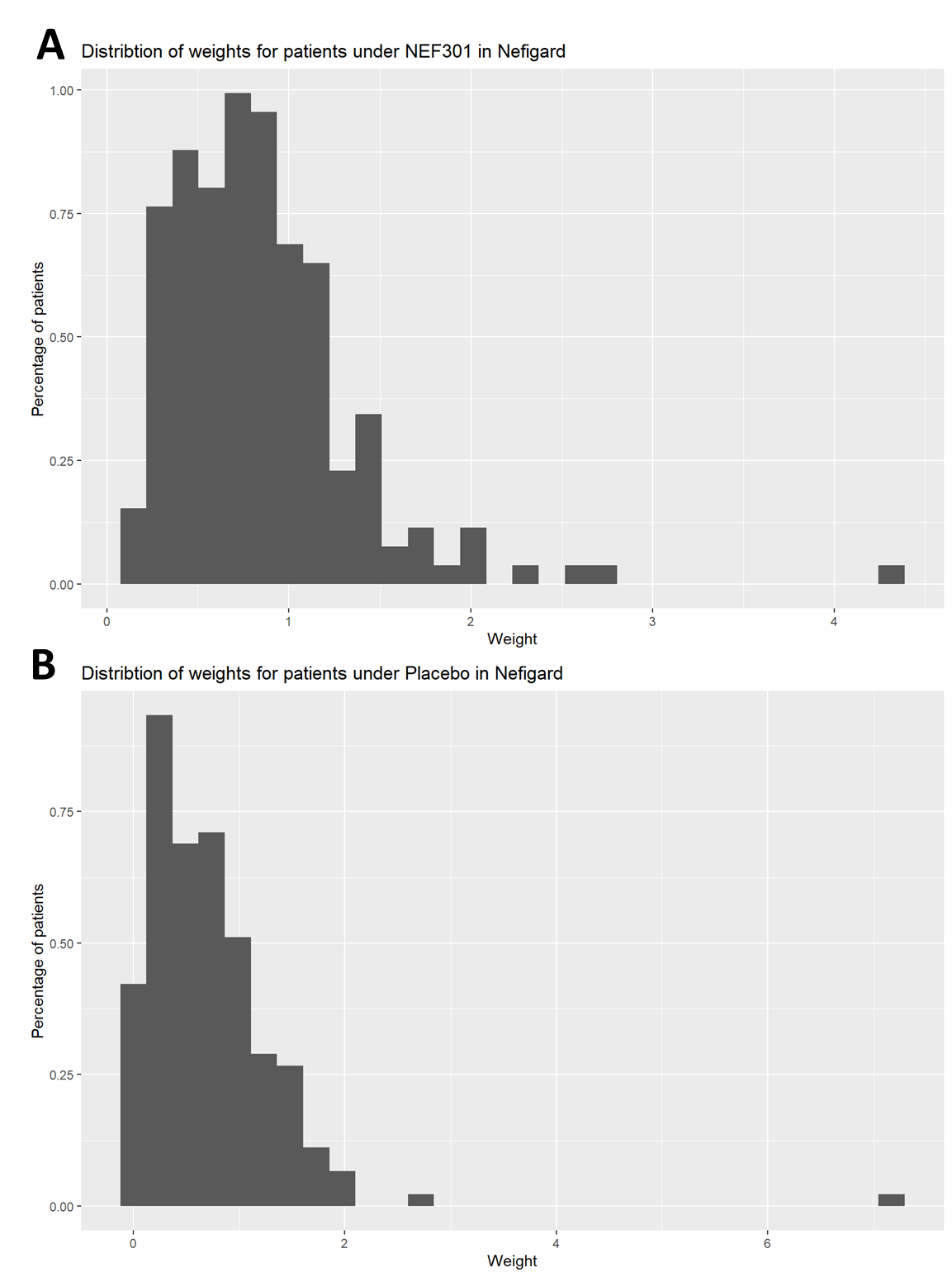


**Supplementary Figure S2.** **Distribution of weights with nefecon (A) and placebo (B) in NefIgArd, derived for the unanchored MAIC.**

MAIC: matching-adjusted indirect comparison.

**A**


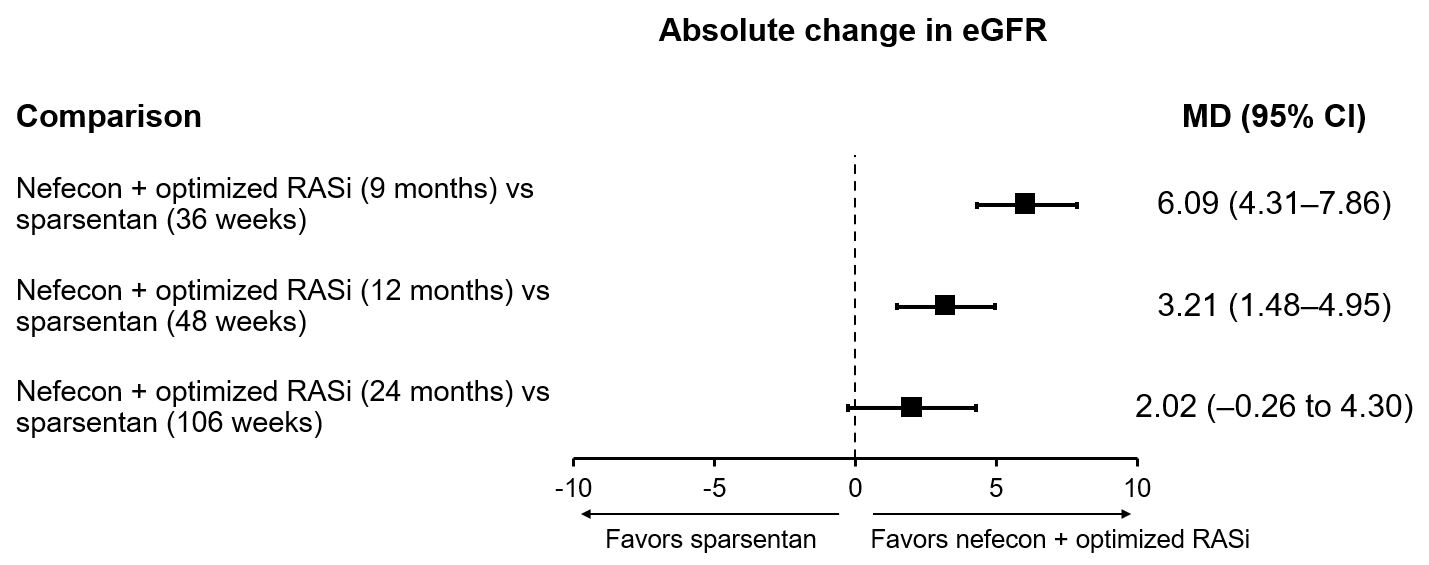


**B**

**
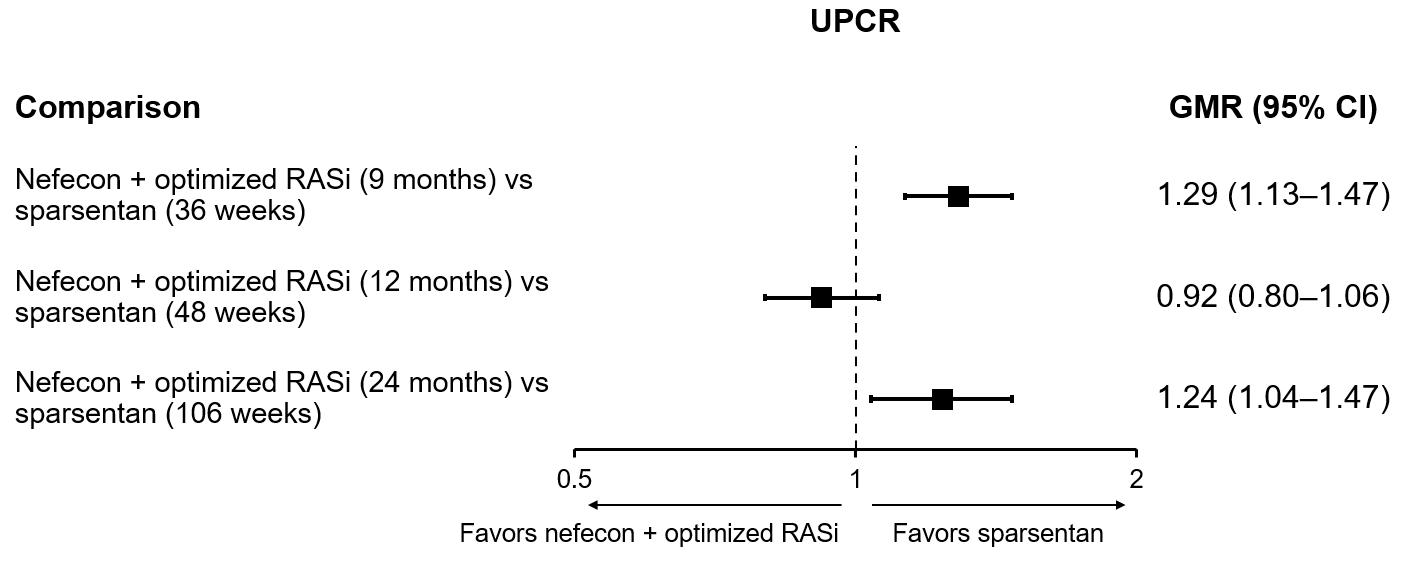
**

**C**

**
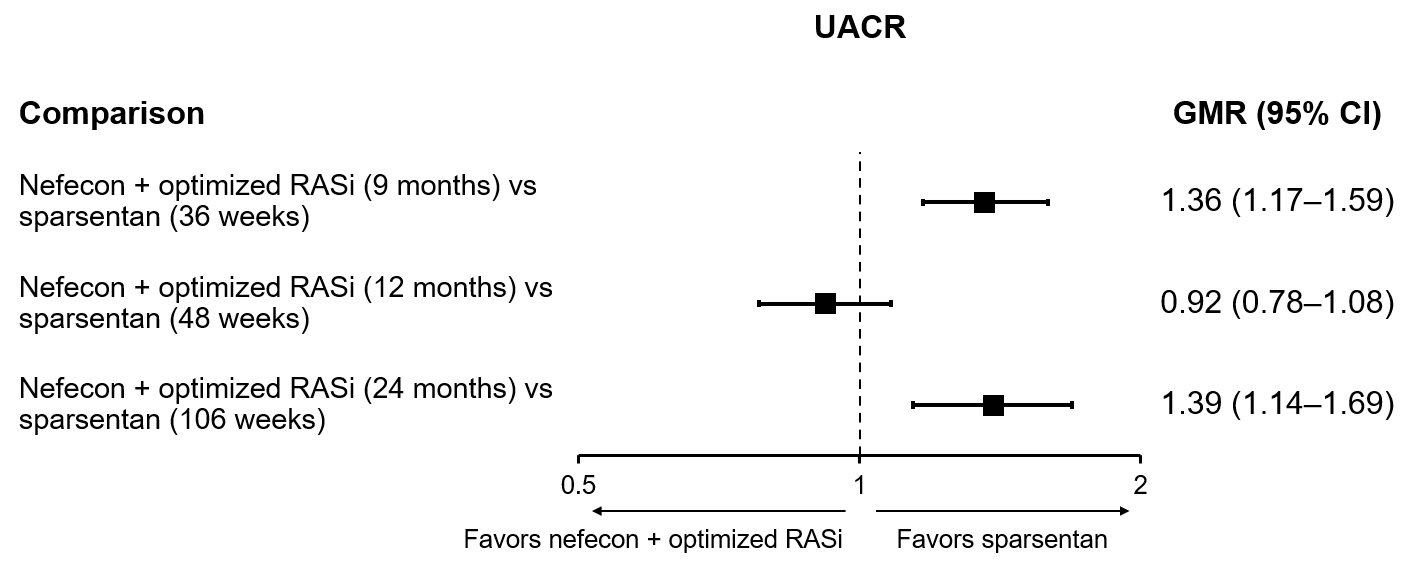
**

**D**


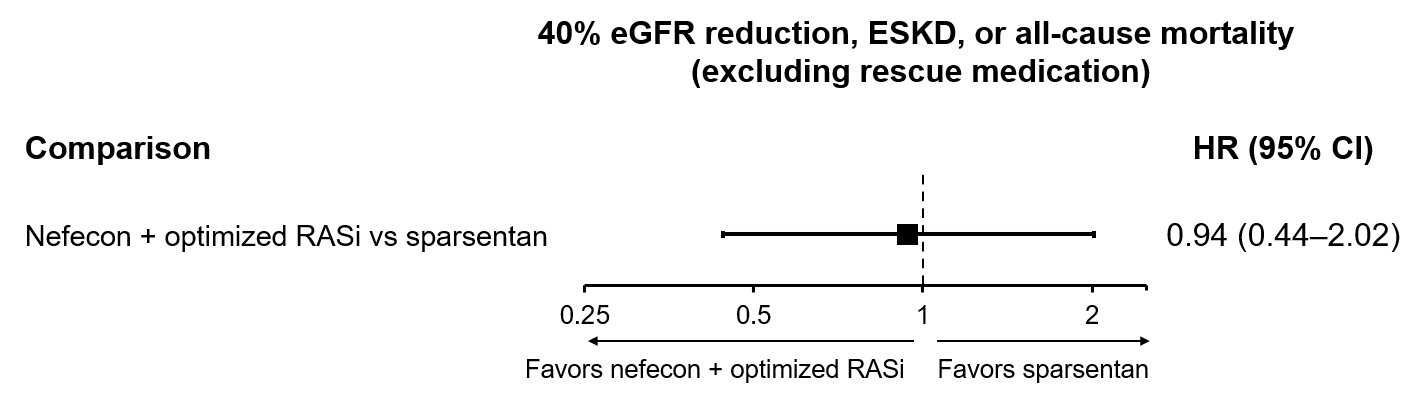


**Supplementary Figure S3.** **Forest plot for the unanchored MAIC for eGFR (A), UPCR (B), and UACR (C) at different time points; forest plot for the unanchored MAIC for time to confirmed 40% eGFR reduction, ESKD, or all-cause mortality (D).**

CI: confidence interval; eGFR: estimated glomerular filtration rate; ESKD: end-stage kidney disease; GMR: geometric mean ratio; HR: hazard ratio; MAIC: matching-adjusted indirect comparison; MD: mean difference; UACR: urine albumin-to-creatinine ratio; UPCR: urine protein-to-creatinine ratio.

**Supplementary References**

1. Caldwell DM, Ades AE and Higgins JP. Simultaneous comparison of multiple treatments: combining direct and indirect evidence. *BMJ*. 331(7521), 897–900 (2005).

2. Rücker G. Network meta-analysis, electrical networks and graph theory. *Res Synth Methods*. 3(4), 312–324 (2012).

3. van Valkenhoef G, Lu G, de Brock B *et al.* Automating network meta-analysis. *Res Synth Methods*. 3(4), 285–299 (2012).

4. van Valkenhoef G, Dias S, Ades AE and Welton NJ. Automated generation of node-splitting models for assessment of inconsistency in network meta-analysis. *Res Synth Methods*. 7(1), 80–93 (2016).

5. Lafayette R, Kristensen J, Stone A *et al.* Efficacy and safety of a targeted-release formulation of budesonide in patients with primary IgA nephropathy (NefIgArd): 2-year results from a randomised phase 3 trial. *Lancet*. 402(10405), 859–870 (2023).

6. Rovin BH, Barratt J, Heerspink HJL *et al.* Efficacy and safety of sparsentan versus irbesartan in patients with IgA nephropathy (PROTECT): 2-year results from a randomised, active-controlled, phase 3 trial. *Lancet*. 402(10417), 2077–2090 (2023).

7. Bensink M, Gong W, Chai X *et al.* Matching-adjusted indirect comparison of sparsentan vs. delayed-release formulation budesonide for proteinuria reduction in adults with IgA nephropathy. Presented at: *60th European Renal Association Congress*. Milan, Italy, June 15–18 2023 (https://medicalaffairs.travere.com/wp-content/uploads/2023/10/2023_ERA_Bensink_MAIC-Spar-v-Budesodine_Focused-Oral-pdf.pdf)

8. Heerspink HJL, Radhakrishnan J, Alpers CE *et al.* Sparsentan in patients with IgA nephropathy: a prespecified interim analysis from a randomised, double-blind, active-controlled clinical trial. *Lancet*. 401(10388), 1584–1594 (2023).

9. Barratt J, Rovin B, Wong MG *et al.* IgA nephropathy patient baseline characteristics in the sparsentan PROTECT study. *Kidney Int Rep*. 8(5), 1043–1056 (2023).

10. Guyot P, Ades AE, Ouwens MJ and Welton NJ. Enhanced secondary analysis of survival data: reconstructing the data from published Kaplan-Meier survival curves. *BMC Med. Res. Methodol.* 12, 9 (2012).
